# Low early ototoxicity rates for pediatric medulloblastoma patients treated with proton radiotherapy

**DOI:** 10.1186/1748-717X-6-58

**Published:** 2011-06-02

**Authors:** Benjamin J Moeller, Murali Chintagumpala, Jimmy J Philip, David R Grosshans, Mary F McAleer, Shiao Y Woo, Paul W Gidley, Tribhawan S Vats, Anita Mahajan

**Affiliations:** 1 Department of Radiation Oncology, University of Texas M.D. Anderson Cancer Center, Houston, TX, USA; 2 Texas Children's Cancer Center, Baylor College of Medicine, Houston, TX, USA; 3 Department of Radiation Oncology, University of Louisville, Louisville, KY, USA; 4 Department of Head and Neck Surgery, University of Texas M.D. Anderson Cancer Center, Houston, TX, USA; 5 Department of Pediatrics, University of Texas M.D. Anderson Cancer Center, Houston, TX, USA

**Keywords:** Proton, radiotherapy, pediatric, medulloblastoma, ototoxicity

## Abstract

**Background:**

Hearing loss is common following chemoradiotherapy for children with medulloblastoma. Compared to photons, proton radiotherapy reduces radiation dose to the cochlea for these patients. Here we examine whether this dosimetric advantage leads to a clinical benefit in audiometric outcomes.

**Methods:**

From 2006-2009, 23 children treated with proton radiotherapy for medulloblastoma were enrolled on a prospective observational study, through which they underwent pre- and 1 year post-radiotherapy pure-tone audiometric testing. Ears with moderate to severe hearing loss prior to therapy were censored, leaving 35 ears in 19 patients available for analysis.

**Results:**

The predicted mean cochlear radiation dose was 30 ^60^Co-Gy Equivalents (range 19-43), and the mean cumulative cisplatin dose was 303 mg/m^2 ^(range 298-330). Hearing sensitivity significantly declined following radiotherapy across all frequencies analyzed (*P *< 0.05). There was partial sparing of mean post-radiation hearing thresholds at low-to-midrange frequencies and, consequently, the rate of high-grade (grade 3 or 4) ototoxicity at 1 year was favorable (5%). Ototoxicity did not correlate with predicted dose to the auditory apparatus for proton-treated patients, potentially reflecting a lower-limit threshold for radiation effect on the cochlea.

**Conclusions:**

Rates of high-grade early post-radiation ototoxicity following proton radiotherapy for pediatric medulloblastoma are low. Preservation of hearing in the audible speech range, as observed here, may improve both quality of life and cognitive functioning for these patients.

## Background

Hearing loss is an important consequence of therapy for children with intracranial malignancies, including medulloblastoma [1,2]. It can have a profound impact on a child's quality of life, affecting not only communication skills but also social and cognitive development [3-5].

Chemotherapy and radiotherapy are major causes of ototoxicity for children with medulloblastoma [6,7]. Efforts to mitigate treatment-related ototoxicity for these patients tumors have included the use of conformal radiotherapy techniques to minimize radiation dose to the auditory apparatus. Compared to conventional photon-based radiotherapy techniques, IMRT reduces cochlear radiation doses and improves both early and late audiometric outcomes [8-10]. Dosimetric studies have suggested that proton techniques can further reduce radiation dose to the auditory apparatus [11-13]. However, whether this translates into a clinical benefit is as yet unknown.

Although ototoxicity is typically considered to be a late effect of radiotherapy, with a latency of approximately four years [14,15], radiation also potentiates early cisplatin-induced ototoxicity when the two are delivered concomitantly [6,7], an effect typically peaking within a year of treatment [8]. The objective of this study is to determine whether proton radiotherapy technique spares this early ototoxicity for children with medulloblastoma.

## Methods

### Patients

Between 2006 and 2009, twenty-three consecutive children with resected and histologically-confirmed medulloblastoma were enrolled on a prospective IRB-approved institutional observational study investigating the effects of proton radiotherapy on normal tissues. Relevant baseline clinicopathologic and demographic features are listed in Table 1.

**Table 1:** Clinical and treatment characteristics

		PROTON COHORT (n = 19)
**Age**		6 (3-16)

**Time to Audiogram (months)**		11 (8-16)

**Gender**	Male Female	14 (74) 5 (26)

**Risk Grouping**	Standard High	16 (84) 3 (16)

**Cisplatin Dose (mg/m^2^)**		303 (298-330)

**CSI Dose (CGE or Gy)**	SR HR	23.4 36.0

**Total Dose (CGE or Gy)**		54.0 or 55.8

**Cochear Dose (CGE or Gy)**		30 (19-43)

Mean values are shown, with data ranges or percentages of total in parentheses. "Time to Audiogram" refers to the interval, in months, between the end of radiotherapy and audiometry. SR = standard-risk, HR = high-risk, CSI = craniospinal irradiation.

### Treatment

All patients received proton-based adjuvant radiotherapy. Patients were positioned supine, and anesthesia was used when necessary to optimize immobilization, at the discretion of the treating physician. CT simulation was performed for each patient (LightSpeed RT16, GE Healthcare). Treatment planning was performed using commercial software (Eclipse, version 8, Varian Medical Systems). Clinical target volumes were defined by the treating physician, and planning margins were calculated as previously described [16-18]. Standard-risk patients (n = 17) received craniospinal irradiation (CSI) to a dose of 23.4 ^60^Co-Gy Equivalents (CGE); high-risk patients (n = 6) received CSI to 36 CGE. The tumor bed, plus a clinical target volume expansion, was boosted to a total dose of between 54 and 55.8 CGE. Relevant details regarding radiation targets and doses are included in Table 1. All patients received platinum-based chemotherapy, with a median cumulative cisplatin dose of 303 mg/m^2 ^(range 298-330 mg/m^2^). All but five patients received adjuvant chemotherapy following radiotherapy; the remainder received it beforehand principally to delay cranial irradiation. The mean total duration of all chemotherapy and radiotherapy was approximately 28 weeks. A chart review confirmed that no other ototoxic drugs were in use by any patient at the time they were simulated for radiotherapy.

### Audiometry

Pure-tone audiometry was performed for each patient at baseline and at 1 year post-radiotherapy. Age-appropriate audiometric techniques were used, at the discretion of the testing audiometrist. Each patient was confirmed free of middle ear disease by tympanometry, in both ears and at both time points. Each audiogram reported hearing threshold, in decibels (dB), for each ear at 0.5, 1, 2, 4, 6, and 8 kHz. Ears with moderate-to-severe hearing loss prior to any therapy were censored. For the remaining patients, Brock ototoxicity rates (Table 2) were determined for each patient from the raw post-radiation audiometric data [19,20]. Ototoxicity rates were calculated per patient and, in the uncommon cases where threshold loss was asymmetric following radiation, toxicity grading reflected the worse of the two ears tested.

**Table 2:** Brock ototoxicity grading scale

FREQUENCY (kHz)	GRADE
-	0

**8**	1

**4**	2

**2**	3

**1**	4

On this scale, ototoxicity is graded by the lowest frequency level at which a hearing threshold loss of at least 40 dB occurs [19]. If no threshold loss of this magnitude is detected at or below 8 kHz, the toxicity grade is zero.

### Radiation Dosimetry

Both cochleae were contoured for each case, and the treatment planning software (Eclipse, version 8, Varian Medical Systems) was used to estimate the mean and maximum delivered organ doses.

### Statistics

Changes in raw audiometric thresholds following radiotherapy were tested for significance by one-way ANOVA (SPSS, version 16). Associations between clinical, demographic, treatment, and audiometric variables were estimated using Spearman's correlations and univariate linear modeling (SPSS, version 16).

## Results

Of the twenty-three patients enrolled, baseline audiometry showed that four had bilateral and three had unilateral severe hearing loss before starting radiotherapy (ototoxicity grades 3 or 4). Of those with bilateral baseline severe hearing loss, two had prior chemotherapy and two had hearing loss attributed to unrelated genetic syndromes. All three patients with unilateral severe hearing loss developed the deficit either before or immediately following surgery, with no prior exposure to chemotherapy or radiotherapy. These ears were censored from analysis, leaving 35 ears in 19 patients available for further study. Baseline demographics were similar to those of most children with medulloblastoma treated at the authors' institution (Table 1).

**Figure 1:**
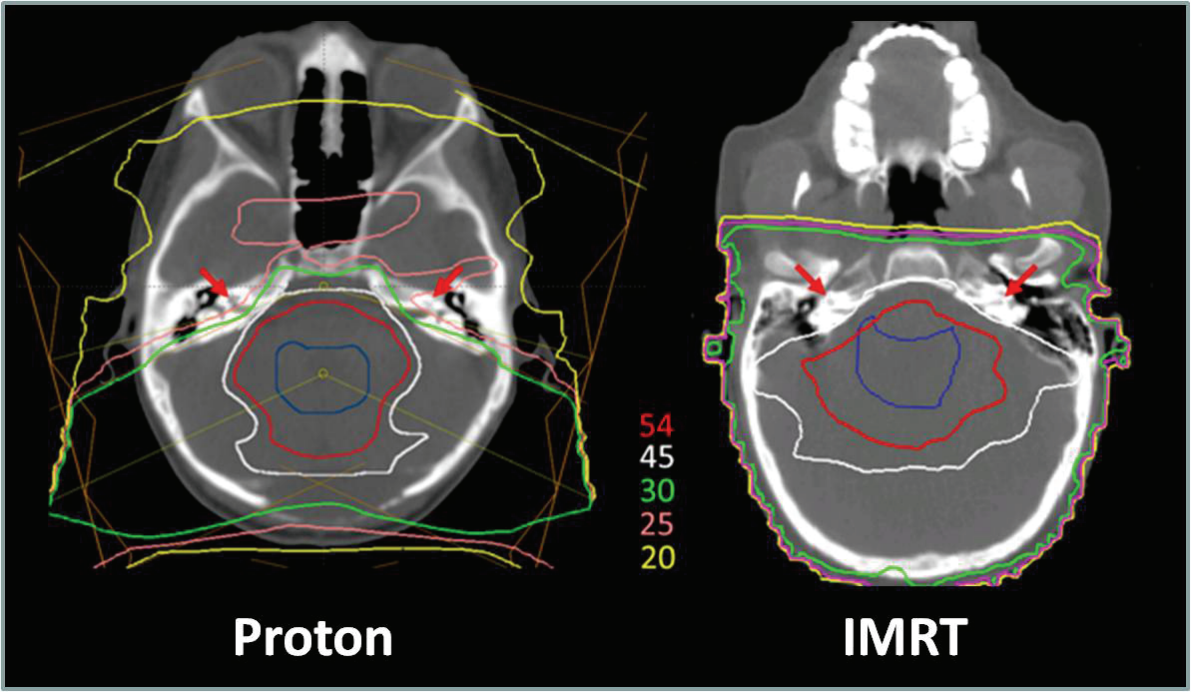
**Proton radiotherapy dosimetry**. A representative plan is shown depicting the sparing of dose to the auditory apparatus (red arrows) in a child with medulloblastoma treated with proton technique. Colored isodose curves are shown depicting the absolute radiation dose in CGE. The clinical tumor bed boost target volume is outlined (blue).

A pair of posterior oblique proton beams was used for the cranial portion of each patient's treatment in order to spare the lenses of the eye while adequately covering the cribriform plate. The auditory apparatus was not typically included as a target volume during the craniospinal portion of treatment. The tumor bed boost portion of treatment was typically carried out using a cone-down postero-lateral beam pair (Figure 1). Consistent with prior reports, proton technique resulted in a favorably low mean cochlear radiation (30 ^60^Co-Gy Equivalents [range 19-43]).

**Figure 2:**
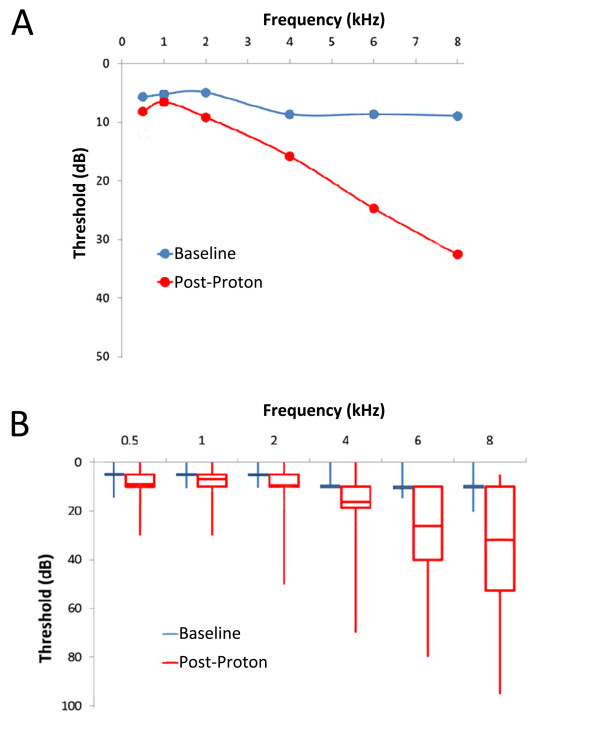
**Audiometric outcomes**. (A) Mean pure-tone audiometry for the proton cohort at baseline (blue) and following radiotherapy (red) are shown. Note the sparing of threshold loss following proton radiotherapy in the audible speech range (0.5-4 kHz). (B) Box and whisker plots of the same data are shown, representing the 2^nd^/3^rd ^quartile data range (boxes), the mean values (horizontal line), and the total data range (whiskers).

**Figure 3:**
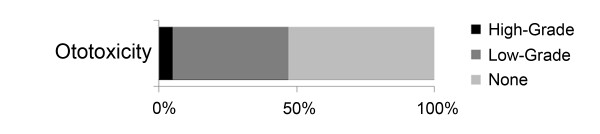
**Ototoxicity rates**. Brock ototoxicity rates, per patient, were favorable following proton radiotherapy (High-Grade = Grades 3 or 4, Low-Grade = Grades 1 or 2, None = Grade 0).

Compared to baseline testing, post-radiation audiometry showed a clinically and statistically significant worsening of hearing threshold across all frequencies tested (*P *< 0.05, Figure 2). However, we noted a relatively modest threshold change in the audible speech range (0.5-6 kHz). The preservation of hearing in the audible speech range is of critical functional importance for patients, and this is reflected in the heavy weighting of threshold loss in this range on ototoxicity grading scales. Accordingly, overall ototoxicity grade was found to be low following proton-based treatment (Figure 3), and the rate of high-grade ototoxicity was favorable at 5%. In keeping with the low rates of high-grade ototoxicity for this cohort, hearing amplification was recommended for only a relatively small number of patients (3 of 19) following radiotherapy.

**Figure 4:**
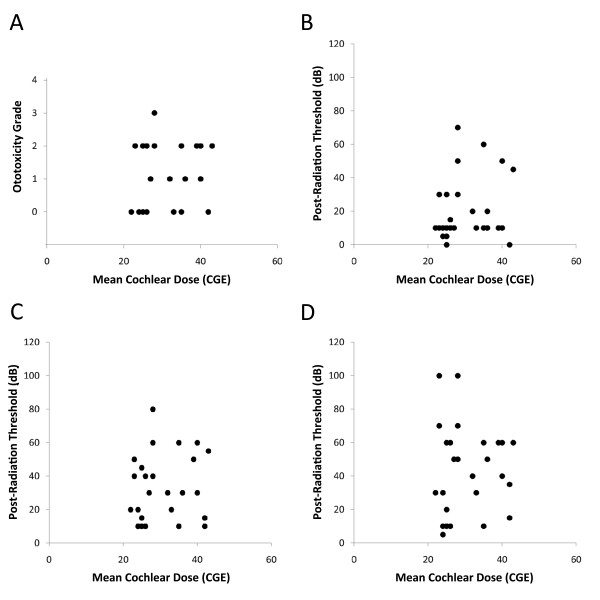
**Dose-response analysis**. Shown are scatter plots of mean predicted cochlear radiation dose versus ototoxicity grade (A), as well as post-proton radiotherapy hearing threshold at 4 kHz (B), 6 kHz (C), and 8 kHz (D). Correlations are weak for all metrics, suggesting a lack of influence of cochlear radiation dose on ototoxicity rates over the range of doses seen in this cohort.

Prior published data suggest that the risk of ototoxicity is linearly related to cochlear radiation dose, with an apparent lower-limit threshold at approximately 36 Gy [15]. If this is the case, then reducing cochlear radiation dose to 36 Gy should minimize ototoxicity, but further reduction of dose below 36 Gy should have little additional impact on hearing loss. Our data support this hypothesis. The audiometric benefits described above for this cohort likely reflect the fact that 84% (16 of 19) of these patients received cochlear doses below 36 CGE; however, we found no evidence that further reducing the cochlear radiation dose below 36 CGE offered any additional benefit to these patients. Although there was a weakly positive correlation between the two (Spearman's ρ = 0.33), radiation dose to the cochlea across the observed range (16-43 CGE) ultimately failed to predict ototoxicity on univariate analysis for these patients. Similarly, scatter plots of cochlear radiation dose versus ototoxicity revealed no obvious correlation between the two (Figure 4). This supports the concept of there being a threshold effect for radiation dose to the cochlea near 36 Gy, and suggests that further reduction in dose below this threshold is unlikely to achieve additional clinical benefit. Of note, cisplatin dose also failed to predict ototoxicity for this cohort, though this is not surprising given the small range of cumulative doses delivered (298-330 mg/m^2^).

## Discussion

The above data support our hypothesis that children with medulloblastoma treated with proton radiotherapy have low rates of ototoxicity at one year after treatment. These data validate the many pre-existing dosimetry studies suggesting that proton technique spares radiation dose to the auditory apparatus, and establish a relationship between this dosimetric advantage and improved clinical outcomes.

To date, published data on audiometric outcomes following proton-based radiotherapy for pediatric medulloblastoma are lacking. Physicians from the Francis H. Burr Proton Center at the Massachusetts General Hospital recently presented in abstract form their early audiometric results in 31 children with medulloblastoma treated with proton radiotherapy [21]. Predicted mean cochlear doses were identical to those for our cohort (30 CGE). At a mean follow-up of 2.5 years, the authors reported high-grade ototoxicity rates of 8% (when correcting for baseline rates). Although this rate is slightly higher than that reported here, the difference is likely related to a higher cumulative cisplatin dose for this cohort (395 versus 303 mg/m^2^) as well as longer follow-up (2.5 versus 1 year). These results corroborate our findings and further support our conclusions that early audiometric outcomes following chemoradiotherapy for children with medulloblastoma are favorable with proton technique.

An unanswered question raised by these results is whether ototoxicity rates following proton therapy are better than those seen following photon therapy. Given the many proposed benefits of proton radiotherapy for pediatric cancer patients, it is unlikely that randomized trials of proton versus photon radiation techniques will ever be pursued in this population. This limits our capacity to make definitive judgments on outcomes between the two techniques. In the absence of higher-quality data, we are left to contrast results across series of patients treated with proton versus photon techniques. We acknowledge that such comparisons are susceptible to many sources of bias and error, and should be interpreted accordingly.

One useful series for comparison is that published by Huang et al [9], which reported early audiometric outcomes after IMRT for children with medulloblastoma. These data demonstrate a higher rate of grade 3-4 toxicity following IMRT (18%) compared to that seen following proton radiotherapy on our study (5%). When comparing the mean post-radiation audiometric data between the two cohorts, there appears to be a sparing of threshold loss following radiation of approximately 10 dB in the audible speech frequency range (1-4 kHz), but little noticeable difference in outcomes between the two modalities at higher or lower frequency ranges.

A potential flaw in this comparison, however, is that the definition of target volume is discrepant between the cohorts. There has been increasing interest recently in reducing the target volume for the boost portion of radiotherapy for children with medulloblastoma to the surgical cavity, alone, without boosting the entire posterior fossa. Accordingly, the entire posterior fossa was targeted only during the craniospinal portion of treatment for the proton patients described above; in the series published by Huang et al [9], the entire posterior fossa was treated to 36 Gy prior to a cone-down boost to the surgical cavity. It is possible that this difference in planning approach, alone, might explain the improvement in audiometric outcomes between the cohorts.

A more robust comparator, then, may be the cohort of IMRT-treated children with medulloblastoma recently reported by Polkinghorn et al [10]. The target volumes and doses for the majority of the patients treated in this cohort were identical to those in our own (23.4 Gy CSI, 55.8 Gy boost). At a median follow-up of 19 months, the reported rate of grade 3-4 hearing loss was similar to that for our cohort (6%). However, whereas more than half of the proton-treated patients on our cohort had no measurable ototoxicity (i.e. grade 0), this was achieved in less than a quarter of the IMRT-treated patients in the comparator cohort. Since low-grade ototoxicity can have an impact on a child's communication skills, learning, and quality of life, this might represent a clinically meaningful benefit to proton radiotherapy for these patients.

Again, however, one must be cautious when drawing conclusions from these comparisons. The delivered doses of cisplatin were not reported in the series published by Polkinghorn et al, so it is not possible to determine whether the cohorts were similar in this regard. Also, though the target volumes for this cohort of IMRT-treated patients were smaller than those for the IMRT-treated patients published by Huang et al, the reported delivered doses to the auditory apparatus were similar (38 Gy versus 37 Gy). Therefore, there may be radiation-unrelated differences between these two IMRT cohorts that account for their divergent audiometric outcomes.

As discussed above, compared to the mean audiometric data available for patients treated with IMRT technique [9], our data show a selective sparing of hearing threshold loss in the audible speech frequency range with proton therapy. This outcome is of particular importance for young radiotherapy patients who are critically reliant on the proper recognition and processing of speech for cognitive and social development. The frequency range quoted for audible speech varies somewhat in the literature, but is most commonly defined as 0.5 to 2 kHz. Recent work has shown that somewhat higher frequency ranges, including 4 kHz, are also quite important for the proper recognition of certain nuances in spoken language, such as fricative sounds, suggesting that our defined range for audible speech ought to be expanded to include these frequencies [5,22]. The observed reduction in the rate of clinically significant threshold loss (i.e. beyond 20 dB) in this range may eventually translate into an improved quality of life for these patients; further follow-up is required to explore this hypothesis.

The established literature shows that radiation dose to the cochlea is clearly an important variable in demonstrating ototoxicity [15]. Though we were unable to demonstrate a dose-response relationship for ototoxicity on our study, this is not surprising given that the vast majority of the patients in our cohort received predicted cochlear doses below the 36 Gy threshold proposed by the existing literature. Indeed, the fact that overall ototoxicity rates were so low on this study supports the validity of dose constraints for the cochlea at or around 36 Gy. Proton radiotherapy effectively allows this constraint to be met in the majority of cases, adding to the overall rationale for its use in this patient population.

However, our results also highlight the importance of variables apart from radiation and chemotherapy dose in determining ototoxicity rates for these patients. Within the relatively narrow range of cisplatin and cochlear radiation doses delivered here, ototoxicity varied widely. High-grade ototoxicity was observed following cochlear radiation doses as low as 28 CGE, much lower than the putative threshold dose. These facts point to the importance of ototoxic variables unrelated to radiation in these patients. As added proof of this concept, the number of enrolled cases censored in this study for having pre-therapy high-grade ototoxicity was higher than the number of analyzed cases with post-radiation high-grade ototoxicity. Further study is needed to better understand the patient and non-therapy related variables that lead to high-grade ototoxicity in some of these children. One such variable may be increased intracranial pressure or, its surrogate, the use of cerebrospinal fluid shunting [14]; however, this factor was not predictive in our cohort. The lack of clear correlations between ototoxicity and these clinical variables may speak to the importance of unidentified biologic factors that may influence individual patients' intrinsic sensitivity to radiation and/or cisplatin effects on the cochlea; such issues warrant further investigation.

Continued follow-up of this cohort is needed for several reasons. First, it will be critical to determine whether the measured clinical gain seen here translates into a benefit in the quality of life for the patients. As the absolute number of patients spared high-grade toxicity was relatively small, answering this question may eventually require a larger sample size. It will also be important to determine whether proton radiotherapy spares late ototoxicity, as this may be a more critical determinant of long-term functional outcomes for these patients than is early toxicity. It seems logical to predict that it will. First, though the data are inherently limited, updates of prior studies have confirmed the stability of early audiometric outcomes with long follow-up for children with medulloblastoma [8,9], and there is no reason to expect that our cohort will behave differently. In fact, it may be that the advantages in proton-treated children will become more pronounced with time, owing to the smaller dose per fraction delivered to the auditory apparatus with proton radiotherapy.

There are some strengths and limitations of this study that should be highlighted. The prospective collection of pre- and post-radiation audiometry on an institutional protocol makes the quality of this dataset favorable in comparison to many of the retrospective reports currently available on this topic. The relative homogeneity of treatment between patients also improves the quality of the data analysis. Also, this is the first series reporting audiometric outcomes for children with medulloblastoma treated with proton radiotherapy and, therefore, it represents a unique contribution to the literature.

Weaknesses of this study include the lack of long-term follow-up and the relatively small sample size of the patient population. For the various reasons outlined in the Introduction, we believe an analysis of audiometric data at one year after radiotherapy is valid. The small sample size is, of course, an inherent obstacle when studying a rare disease treated with a limited resource. Continued accrual onto prospective studies of normal tissue toxicity is critical to further evaluate the proposed benefits of proton radiotherapy for children with medulloblastoma and other malignancies.

It could be argued that omitting the high-risk patients from each cohort would have improved the homogeneity of the populations compared. While this may be true, this approach also would have carried with it the drawbacks of decreasing the cohort size, reducing the range of the radiation dose dataset, and decreasing the scope of the study. Indeed, repeating the major analyses described above while including only the standard-risk patients had no noticeable impact on the data, other than by decreasing the mean radiation dose delivered to the cochlea (not shown). Therefore, inclusion of these patients in the above analyses appears to be appropriate.

## Conclusions

Proton radiotherapy results in low early high-grade ototoxicity rates for children with medulloblastoma. The sparing of auditory threshold in the audible speech range with proton radiotherapy may eventually translate into improved communication skills, quality of life, social development, and cognitive development for these patients. Further follow-up is needed to address these questions, and to determine the degree to which proton technique may prevent late ototoxicity.

## Competing interests

The authors declare that they have no competing interests.

## Authors' contributions

BJM designed the study, analyzed the data and prepared the manuscript. JJP collected and helped to analyze the data. MC, DRG, MFM, SYW, PWG, TSV, and AM all participated in the treatment of the patient cohort described, designed the study, helped analyze the data and assisted with preparation of the manuscript. All authors read and approved the final manuscript.
